# Stabilization and quantitative measurement of nicotinamide adenine dinucleotide in human whole blood using dried blood spot sampling

**DOI:** 10.1007/s00216-022-04469-7

**Published:** 2022-12-11

**Authors:** Ryo Matsuyama, Tomoyo Omata, Michiharu Kageyama, Ryota Nakajima, Masanobu Kanou, Kei Yamana

**Affiliations:** 1 Nutraceutical Group, Healthcare New Business Division, Teijin Limited, Hino, Tokyo Japan; 2 Discovery DMPK Research Group, Toxicology & DMPK Research Department, Teijin Institute for Bio-Medical Research, Teijin Pharma Limited, Hino, Tokyo Japan; 3 NOMON Co. Ltd., Kasumigaseki, Chiyoda-Ku, Tokyo Japan

**Keywords:** Dried blood spot, Nicotinamide adenine dinucleotide, Nicotinamide mononucleotide, Storage stability

## Abstract

Nicotinamide adenine dinucleotide (NAD^+^) is a coenzyme essential for energy production. Recently, associations between NAD^+^ and aging-related diseases have been reported, and NAD^+^ precursors that increase NAD^+^ concentration in the body have been acknowledged as anti-aging supplements. However, there have been only a few studies on the link between aging or aging-related diseases and human blood NAD^+^ concentration because NAD^+^ and its precursors are unstable in blood and difficult to measure. Therefore, we aimed to construct a quantitative NAD^+^ measurement method that is simpler than the existing methods. The calibration standards of NAD^+^ showed good linearity (0.9936 to 0.9990) in the range of 0.25 to 200 μM, and the lower limit of quantification was 0.5 to 2 μM. We found that QIAcard FTA DMPK-B maintained NAD^+^ stability of 85% or more for at least 2 weeks at 4 °C and 1 week at room temperature using the dried blood spot method. Additionally, NAD^+^ stability in the blood extraction solution was more than 90% for 2 months. To our knowledge, there has been no report on a quantitative NAD^+^ measurement method in human whole blood that can be performed with as little as 5 μL of blood and can be easily implemented at both medical clinics and private homes. Our simple and convenient method has the potential to become the gold standard for NAD^+^ measurement in blood. It is expected to contribute to the acceleration of research on the correlation between aging or aging-related diseases and NAD^+^ concentration in human blood.

**Supplementary Information:**

The online version contains supplementary material available at 10.1007/s00216-022-04469-7.

## Introduction

Nicotinamide adenine dinucleotide (NAD^+^) is a coenzyme essential for energy production. With aging, the concentration of NAD^+^ decreases in tissues in rodents and humans [1–4]. In addition, NAD^+^ precursors, such as nicotinamide mononucleotide (NMN) and nicotinamide riboside (NR), increase the concentration of NAD^+^ in the tissues [5, 6] and are considered to ameliorate or prevent aging-related diseases. The concentration of NAD^+^ and its precursors in vivo is expected to serve as a biomarker for pathological conditions and/or aging. However, only a few studies have investigated the correlation between various pathological conditions or aging and NAD^+^ concentration in human blood [7]. This can be attributed to the difficulty associated with NAD^+^ measurement, especially in humans. Sampling and measurements can be performed only in facilities with specialized equipment, as NAD^+^ and its precursors have poor stability in whole blood and red blood cells (RBCs), and sample preparation, involving chemical treatment and rapid freezing, is required for quantification [8–11]. Therefore, it is necessary to establish a stable and simple NAD^+^ measurement method that will enable more frequent measurements.

Dried blood spot (DBS) sampling is a simple sampling method. It involves spotting blood on a special filter paper, drying, extracting, and quantifying the concentration of the substance of interest present in the blood. DBS sampling has the following advantages: (1) it does not require the separation of plasma from blood; (2) it can be performed with a smaller volume of blood than conventional blood tests, allowing self-collection of blood; and (3) the sample can be easily transported to the measurement facility [12–14]. As a simple sampling method, it is often used for newborn mass screening [12], and recently, its application to detect SARS-CoV-2 antibodies is also being considered [15]. However, DBS is sensitive to storage conditions, and the stability of some target substances may be compromised [13]. In this study, we developed a simple blood NAD^+^ quantitative measurement system using the DBS sampling method in order to facilitate human blood NAD^+^ measurement.

## Materials and methods

### Chemicals

Whatman FTA DMPK-A, DMPK-B, and DMPK-C cards were purchased from GE Healthcare UK Ltd. (Little Chalfont, UK). QIAcard FTA DMPK-A, DMPK-B, and DMPK-C cards were purchased from QIAGEN GmbH (Hilden, Germany). The 903 Protein Saver Snap Apart Card was purchased from Global Life Sciences Solutions Operations UK Ltd. (Little Chalfont, UK). β-Nicotinamide adenine dinucleotide-d4 (d4-NAD^+^), β-nicotinamide-d4 mononucleotide (d4-NMN), and β-nicotinamide mononucleotide (NMN) were purchased from Toronto Research Chemicals, Inc. (Toronto, Canada). β-Diphosphopyridine nucleotide (NAD^+^), formic acid, and ammonium formate were purchased from Fujifilm Wako Pure Chemical Corporation (Osaka, Japan). Heparin sodium was purchased from NIPRO Corporation (Osaka, Japan). Acetonitrile was purchased from Thermo Fisher Scientific Inc. (Waltham, MA, USA) and Kanto Chemical Co., Inc. (Tokyo, Japan). Ultra-pure water was obtained using Milli-Q Integral 5 and Milli-Q Gradient A10 (Merck Millipore, Burlington, MA, USA). β-Nicotinamide-d5 mononucleotide (d5-NMN) was synthesized by Teijin Limited (Tokyo, Japan).

### Animal study for the optimization of extraction buffer

Six-week-old male Sprague Dawley rats were purchased from Jackson Laboratory Japan, Inc. (Kanagawa, Japan). The rats were housed under controlled environmental conditions with a 12-h light/dark cycle and free access to water and food. The rats were anesthetized with isoflurane and euthanized by collecting blood from the abdominal aorta. The collected blood was stored on ice until use. A portion of the blood was centrifuged (13,200 × *g*, 5 min, 4 °C) and fractionated into plasma (supernatant) and blood cells (residue). The pelleted erythrocytes were loosened by tapping and pipetting before collection. Blood, plasma, or blood cells (5 μL) were placed in polypropylene tubes; then, 10 μL of aqueous internal standard (IS; 20 μM d4-NAD^+^ and 10 μM d5-NMN) and 295 μL of each extraction buffer with acetonitrile at different concentrations (0%, 20%, 40%, 60%, 80%, and 100%) were added. Samples were mixed using a micromixer (TOMY Microtube Mixer MT-400; TOMY Digital Biology Co., Ltd., Tokyo, Japan) for 30 min at room temperature (20–30 °C). The mixed samples (100 μL) were placed in clean polypropylene tubes with 200 μL of acetonitrile, followed by stirring and centrifugation (24,250 × *g*, 10 min, 4 °C). Water with IS was added to 100 μL of the supernatant after centrifugation to a final organic solvent content of 67%. Thereafter, the samples were analyzed using liquid chromatography-mass spectrometry (LC–MS/MS). All procedures involving animals were conducted in accordance with the guidelines for animal experimentation set by the Animal Ethics Committee of the Teijin Institute for Bio-Medical Research, Teijin Pharma Limited, Tokyo, Japan (approval number: A21-031-R).

### Human blood collection

Human blood (120 μL) was collected from healthy, in-house, adult volunteers via self-collection using the Micro Blood Collection Kit (Micro Blood Science Inc., Tokyo, Japan). The collection was performed multiple times, with five individuals each time. The donors fasted on the morning of the day of blood collection, and blood was collected around 9–10 a.m. The collected blood was stored on ice until use. All procedures were conducted in accordance with the guidelines for life science/medical research targeting humans set by the Human Ethics Committee of the Teijin Institute for Bio-Medical Research, Teijin Pharma Limited, Tokyo, Japan (approval number: HB21-002-M1).

### Preparation of dried blood spots

**Fig. 1:**
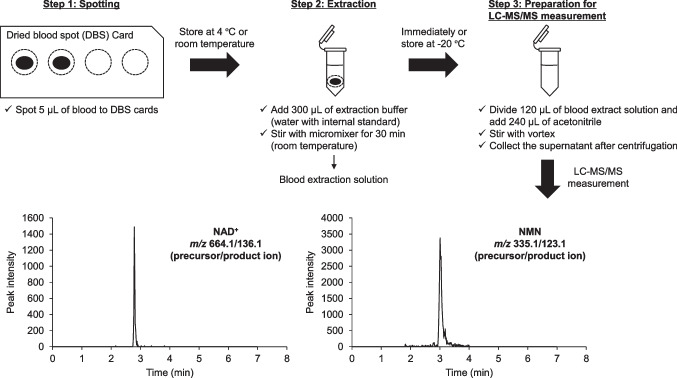
Procedure followed for the quantitative measurement of nicotinamide adenine dinucleotide (NAD^+^) and nicotinamide mononucleotide (NMN) in blood using dried blood spot sampling

The collected human blood samples (5 μL) were spotted on each DBS card, sealed in a standardized zip pouch of MARUTO moisture absorption film (MARUTO SANGYO CO., LTD., Fukuoka, Japan), and stored at 4 °C or at room temperature (20–30 °C). After storage for a certain period of time, the filter paper with entire blood spots was cut with scissors and placed in a polypropylene tube with 300 μL of extraction buffer containing IS (water in 667 nmol/L d4-NAD^+^ and 333 nmol/L d4-NMN). The samples were mixed using a micromixer (TOMY Microtube Mixer MT-400) for 30 min at room temperature to prepare a blood extraction solution. A portion of the blood extraction solution was added to a polypropylene tube with a twofold volume of acetonitrile and stirred. After centrifugation (24,250 × *g*, 10 min, 4 °C), the supernatant was analyzed using LC–MS/MS (Fig. 1). Some samples were immediately extracted after blood spotting to confirm the extraction efficiency of the DBS cards. Moreover, the blood extraction solution or its solution after adding acetonitrile extracted 15 days after refrigerated storage on filter paper was further stored at − 20 °C for 71 days. The blood extraction solution was centrifuged after adding a twofold volume of acetonitrile after storage. These solutions were evaluated for storage stability of NAD^+^/NMN after extraction. For the initial (day 0) sample, 5 μL of blood was directly added to 300 μL of extraction buffer on the day of blood collection without spotting on filter paper, and then mixed with a micromixer to obtain a blood extraction solution. In addition, some collected blood samples were stored at 4 °C or room temperature without any treatment, and then treated in the same manner as described above to evaluate the storage stability of NAD^+^/NMN in whole blood.

### LC–MS/MS analytical method

LC–MS/MS analysis was conducted using the NexeraX2 system (Shimadzu Corporation, Kyoto, Japan) coupled with the 4000 Qtrap system (AB Sciex, Framingham, MA, USA). The samples (3–5 μL) were injected into an Atlantis HILIC Silica column (5 μm, 4.6 mm × 50 mm) (Waters, Milford, MA, USA) and maintained at 50 °C. Elution was conducted at a flow rate of 1 mL/min under a gradient condition of 0.05% (v/v) formic acid containing 10 mmol/L ammonium formate aqueous solution (mobile phase A) and acetonitrile (mobile phase B). The following two types of gradient conditions were used: (1) optimization of the extraction buffer, 0–5 min: 97–40% B, 5–7 min: 40% B, 7–7.1 min: 97% B, and 7.1–10 min: 97% B; (2) other studies, 0–1 min: 80% B, 1–3 min: 80–40% B, 3–4.5 min: 40% B, 4.5–4.6 min: 40–80% B, and 4.6–7 min: 80% B. Multiple reaction monitoring mode (positive ion mode) was used to monitor ions as follows: precursor ion/product ion; NAD^+^ (664.1/136.1), d4-NAD^+^ (668.2/136.1), NMN (335.1/123.1), d4-NMN (339.1/126.9), and d5-NMN (340.1/127.0). As NAD^+^ and NMN are endogenous substances, calibration standards were prepared using water instead of blood at 10 concentrations in the range of 0.25–250 μM (optimization of the extraction buffer) or 0.25–200 μM (other studies).

### Data analysis

LC–MS/MS data were acquired and quantified using Analyst software version 1.6.2 (AB Sciex). The calibration curve acceptance criteria were as follows: the analyte concentration should be ± 15% (except for the lower limit of quantification (LLOQ), ± 20%) of the nominal (theoretical) concentrations, and a minimum of six concentrations should meet the above criteria in each run. The LLOQ varied between runs and ranged from 0.5 to 2 μM for NAD^+^ and from 0.25 to 2 μM for NMN. Correlation coefficients of the calibration standards for NAD^+^ and NMN were greater than 0.99.

Each parameter was calculated as indicated below:

- Storage stability of NAD^+^/NMN in human whole blood (%) = concentration of NAD^+^/NMN after storage / concentration of NAD^+^/NMN before storage (day 0) × 100
- Storage stability of NAD^+^/NMN on DBS cards (%) = concentration of NAD^+^/NMN after storage on DBS cards / concentration of NAD^+^/NMN without cards before storage (day 0) × 100
- Extraction efficiency (%) = concentration of NAD^+^/NMN with cards (day 0) / concentration of NAD ^+^/NMN without cards (day 0) × 100
- Storage stability of NAD^+^/NMN in buffer after extraction (%) = concentration of NAD^+^/NMN in blood extraction solution after storage for 71 days at − 20 °C / concentration of NAD^+^/NMN before storage (day 0) × 100

## Results

### Optimization of extraction buffer using rat whole blood

Rat blood, blood cells, and plasma were treated with each extraction buffer with acetonitrile at different concentrations (0%, 20%, 40%, 60%, 80%, and 100%) to optimize the extraction of NAD^+^ and NMN from each sample. The LC–MS/MS results showed that concentrations of NAD^+^ and NMN in blood were the highest when only water was used as the extract solution, and the concentration decreased considerably when the acetonitrile content was 80% or more (Table 1). This tendency was similar for blood cells, but the concentrations of NAD^+^ and NMN in plasma were below that of the LLOQ in all extraction buffers except for 100% water. Water was used as the extraction buffer in this study because water was found to be the most suitable agent for extracting NAD^+^ and NMN from blood.

**Table 1:** Optimization of extraction buffer using rat whole blood (*n* = 3)

Analyte	Composition of extraction buffer (water:acetonitrile)	Concentration (μM)								
		Whole blood			Blood cells			Plasma		
		Mean	SD	%CV	Mean	SD	%CV	Mean	SD	%CV
NAD^+^	0%:100%	NC	NC	NC	NC	NC	NC	NC	NC	NC
	20%:80%	18.28	3.89	21.3	31.53	6.00	19.0	NC	NC	NC
	40%:60%	124.05	13.98	11.3	203.81	33.78	16.6	NC	NC	NC
	60%:40%	141.35	10.45	7.4	212.89	26.45	12.4	NC	NC	NC
	80%:20%	135.60	25.70	19.0	232.44	22.34	9.6	NC	NC	NC
	100%:0%	200.78	40.22	20.0	380.82	130.19	34.2	1.91	NC	NC
NMN	0%:100%	NC	NC	NC	NC	NC	NC	NC	NC	NC
	20%:80%	NC	NC	NC	NC	NC	NC	NC	NC	NC
	40%:60%	5.54	1.53	27.6	9.96	0.76	7.6	NC	NC	NC
	60%:40%	5.88	0.83	14.1	12.58	0.92	7.3	NC	NC	NC
	80%:20%	6.25	1.20	19.2	12.23	1.03	8.4	NC	NC	NC
	100%:0%	6.45	0.19	2.9	12.03	0.65	5.4	NC	NC	NC

Lower limit of quantification: 1 μM (NAD^+^), 2 μM (NMN)

*NAD*^+^ nicotinamide adenine dinucleotide;

*NMN* nicotinamide mononucleotide;

*NC* not calculated, as the concentrations in most samples were below the limit of quantification (BLQ)

### Concentrations of NAD^+^ and NMN in human whole blood and the storage stability of NAD^+^ and NMN at 4 °C

The NAD^+^ and NMN concentrations (mean ± standard deviation (SD)) in whole blood samples measured on the day of collection (day 0) were 31.89 ± 6.43 and 1.85 ± 0.43 μM, respectively (Table 2). After storing whole blood for different periods after collection, the storage stability of NAD^+^ (mean ± SD) was as follows: 123.7% ± 23.8% (2 days), 134.9% ± 19.8% (1 week), 101.5% ± 17.8% (2 weeks), and 64.4% ± 5.9% (1 month) (Table 3). The storage stability of NMN was 52.1% ± 20.4% (2 days), 27.8% ± NC (not calculated because the concentrations in two of the four samples were below the limit of quantification (BLQ)) (1 week), and 65.7% ± 34.1% (1 month). The concentrations of NMN in most samples were BLQ after storage for 2 weeks.

**Table 2:** Concentration of nicotinamide adenine dinucleotide (NAD^+^) and nicotinamide mononucleotide (NMN) in human whole blood on day 0

Age (years)	n*	Concentration in human whole blood (μM)					
		NAD^+^			NMN		
		Mean	SD	%CV	Mean	SD	%CV
20–29	9	32.39	7.14	22.0	1.85	0.47	25.3
30–39	10	31.99	7.10	22.2	1.80	0.44	24.5
40–49	1	28.61	NC	NC	2.15	NC	NC
50–59	4	31.35	4.94	15.8	1.88	0.42	22.5
All	24	31.89	6.43	20.1	1.85	0.43	23.0

*NC* not calculated as *n* = 1

^*^Total number of people

**Table 3:** Storage stability of nicotinamide adenine dinucleotide (NAD^+^) and nicotinamide mononucleotide (NMN) in human whole blood at 4 °C

Storage period	Storage stability (%)							
	NAD^+^				NMN			
	Mean	SD	%CV	*n*	Mean	SD	%CV	*n*^2)^
2 days	123.7	23.8	19.3	9	52.1	20.4	39.1	9
1 week	134.9	19.8	14.7	4	27.8^1)^	NC	NC	4 (2)
2 weeks	101.5	17.8	17.5	9	NC	NC	NC	9 (6)
1 month	64.4	5.9	9.1	9	65.7	34.1	51.8	9 (3)

Lower limit of quantification: 0.5 to 2 μM (NAD^+^), 0.25 to 1 μM (NMN)

Storage stability (%) = concentration after storage / concentration before storage (day 0) × 100

*NC* not calculated, as the concentrations in most samples were BLQ

^1)^The average value of two samples was calculated because the concentrations in two of the four samples were below the limit of quantification (BLQ)

^2)^Numbers in parentheses represent the number of exclusions based on BLQ

### Storage stability of NAD^+^ and NMN on DBS cards

**Fig. 2:**
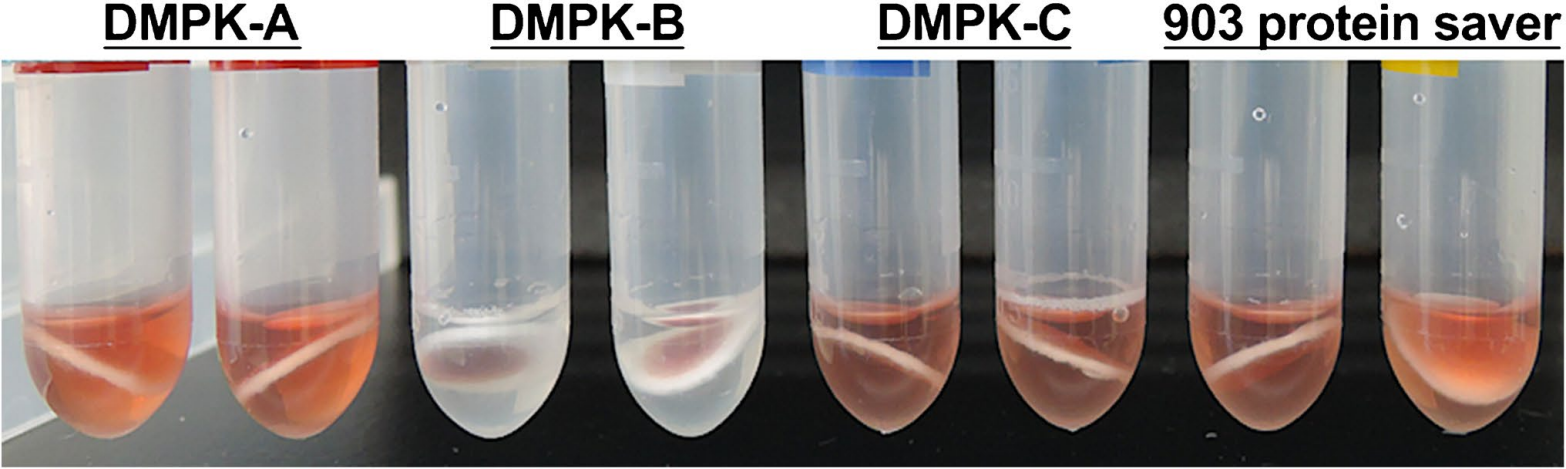
Comparison of blood extraction solutions. The dried blood spot cards were added to the extraction buffer (water with internal standard) and stirred with a micromixer for 30 min at room temperature

**Fig. 3:**
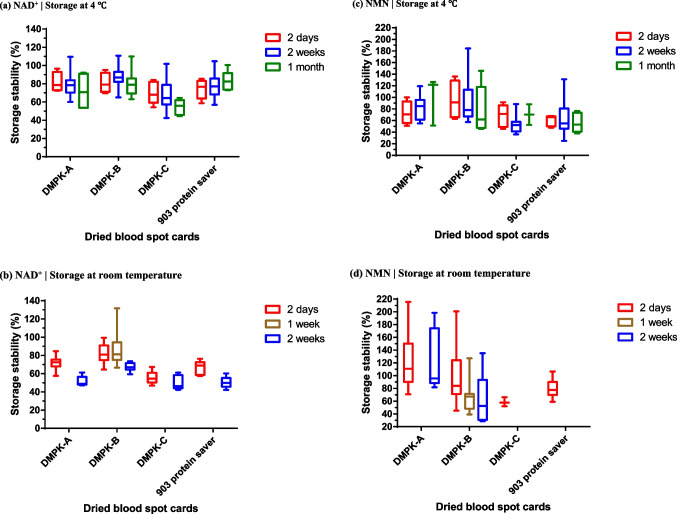
Storage stability of nicotinamide adenine dinucleotide (NAD^+^) (**a**, **b**) and nicotinamide mononucleotide (NMN) (**c**, **d**) on dried blood spot cards. The box extends from the 25th to 75th percentiles, and the whiskers show the smallest and largest values. The line in the middle of the box indicates the median. The graphs were generated using GraphPad Prism 6 for Windows, version 6.07 (GraphPad Software, Inc., San Diego, CA, USA)

Human whole blood was spotted on four types of commercially available DBS cards (Table 4), and the stability of NAD^+^ and NMN after storage at 4 °C and room temperature was evaluated. The blood extraction solution of DMPK-B was transparent, whereas the solution of the other cards was red (Fig. 2). DMPK-B presented the highest NAD^+^ storage stability (mean ± SD), reaching 80.7% ± 11.0% (2 days), 87.8% ± 11.2% (2 weeks), and 79.8% ± 13.9% (1 month) at 4 °C, and 82.0% ± 9.9% (2 days), 87.2% ± 21.4% (1 week), and 67.6% ± 4.6% (2 weeks) at room temperature (Table S1, Fig. 3). Subsequently, the storage stability of NAD^+^ on different cards decreased as follows: DMPK-A > 903 protein saver > DMPK-C. The stability of NAD^+^ after storage at room temperature for 2 days on other DBS cards, except for DMPK-B, was as follows: 71.6% (DMPK-A), 55.9% (DMPK-C), and 66.2% (903 protein saver), which were lower than the stability of NAD^+^ after storage at room temperature for 1 week on DMPK-B. In addition, the storage stability of NAD^+^ on all DBS cards, including DMPK-B, at room temperature for 2 weeks was lower than that at 4 °C. DMPK-B also presented the highest storage stability (mean ± SD) of NMN among all DBS cards, reaching 95.4% ± 33.1% (2 days), 93.6% ± 38.1% (2 weeks), and 78.6% ± 40.2% (1 month) at 4 °C. However, the stability of NMN showed greater variability than that of NAD^+^ on all DBS cards.

**Table 4:** Components of dried blood spot (DBS) cards

DBS card	Material of paper	Impregnated chemicals	Paper thickness (μm)
Whatman FTA DMPK-A (QIAcard FTA DMPK-A)	Cellulose based	Radical inhibitors (sodium dodecyl sulfate, tris (hydroxymethyl)aminomethane)	562
Whatman FTA DMPK-B (QIAcard FTA DMPK-B)	Cellulose based	Chaotropic agents (guanidium thiocyanate)	744
Whatman FTA DMPK-C (QIAcard FTA DMPK-C)	Cotton based	No coating	524
903 protein saver card	Cotton based	No coating	509

Adapted from Lim 2018 [20] and Luckwell et al. 2013 [21]

### NAD^+^ and NMN extraction efficiency of DBS cards

As the storage stability results were obtained using the initial value measured without DBS cards, the results may be affected by not only pure storage stability but also extraction efficiency. Therefore, the extraction efficiency was evaluated using samples extracted from DBS cards immediately after blood spotting. The NAD^+^ extraction efficiency of each DBS card was as follows: 95.1% ± 11.2% (DMPK-A), 90.9% ± 11.4% (DMPK-B), 72.9% ± 18.1% (DMPK-C), and 77.2% ± 19.0% (903 protein saver), indicating that DMPK-A and DMPK-B had high extraction efficacy (Table 5). However, the NMN extraction efficiency was 125.6% ± 61.8% (DMPK-A), 87.7% ± 36.9% (DMPK-B), 87.9% ± 58.6% (DMPK-C), and 73.2% ± 25.2% (903 protein saver), showing large variability compared with NAD^+^ extraction efficiency.

**Table 5:** Extraction efficiency of nicotinamide adenine dinucleotide (NAD^+^) and nicotinamide mononucleotide (NMN) on DBS cards

DBS card	Extraction efficiency (%)							
	NAD^+^				NMN			
	Mean	SD	%CV	*n*	Mean	SD	%CV	*n*^1)^
DMPK-A	95.1	11.2	11.8	9	125.6	61.8	49.2	9
DMPK-B	90.9	11.4	12.5	14	87.7	36.9	42.1	14
DMPK-C	72.9	18.1	24.9	9	87.9	58.6	66.7	9
903 protein saver	77.2	19.0	24.6	9	73.2	25.2	34.4	9 (1)

Extraction efficiency (%) = concentration with cards (day 0) / concentration without cards (day 0) × 100

Lower limit of quantification: 0.5 to 2 μM (NAD^+^), 0.25 to 1 μM (NMN)

^1)^Numbers in parentheses represent the number of exclusions based on below the limit of quantification

### Storage stability of NAD^+^ and NMN after extraction

The NAD^+^ stability in blood extraction solution from DMPK-B was 93.1% ± 8.4% (100% water) and 100.0% ± 16.1% (67% (v/v) acetonitrile water), and NMN stability was 86.3% ± 17.2% (100% water) and 84.1% ± 33.7% (67% (v/v) acetonitrile water), showing good stability during storage in either solution (Table 6). The NAD^+^ stability in blood extraction solution from other DBS cards showed a large increase when stored in 100% water (DMPK-A: 145.8% ± 12.5%, DMPK-C: 200.6% ± 62.0%, 903 protein saver: 266.3% ± 59.6%), but not did not show this tendency during storage in 67% (v/v) acetonitrile water (DMPK-A: 89.6% ± 7.1%, DMPK-C: 73.6% ± 12.6%, 903 protein saver: 92.1% ± 15.1%). In addition, there was no increase in NMN stability.

**Table 6:** Storage stability of nicotinamide adenine dinucleotide (NAD^+^) and nicotinamide mononucleotide (NMN) after extraction

	Storage stability in buffer (%)					
	NAD^+^			NMN		
	Mean	SD	%CV	Mean	SD	%CV
Blood extraction solution (100% water)						
DMPK-A	145.8	12.5	8.6	73.7	15.6	21.1
DMPK-B	93.1	8.4	9.0	86.3	17.2	19.9
DMPK-C	200.6	62.0	30.9	63.7	19.3	30.2
903 protein saver	266.3	59.6	22.4	78.4	17.1	21.8
Blood extraction solution with acetonitrile (67% (v/v) acetonitrile water)						
DMPK-A	89.6	7.1	7.9	84.7	24.3	28.7
DMPK-B	100.0	16.1	16.1	84.1	33.7	40.0
DMPK-C	73.6	12.6	17.1	32.7	5.0	15.3
903 protein saver	92.1	15.1	16.4	52.4	15.1	28.8

Storage stability in buffer (%) = concentration in blood extraction solution after storage for 71 days at − 20 °C / concentration before storage (day 0) × 100

The blood extraction solution was extracted from DBS cards after storage for 2 weeks at 4 °C

## Discussion

As NAD^+^ in whole blood or RBCs is unstable at room temperature, it is important to store samples for NAD^+^ measurement at a low temperature as quickly as possible. Furthermore, there are reports regarding the solvent added during storage [8–11]. In our study, the optimum extraction buffer for NAD^+^ and NMN was water, whereas the buffer containing acetonitrile at high concentrations showed lower extraction efficiency than water (Table 1). NAD^+^ and NMN were not detected in plasma, but were detected in blood cells, indicating that they are mainly localized in blood cells. Therefore, water is considered the best extraction buffer owing to the high burst effect of blood cells (hemolysis) from the osmotic pressure. Conversely, organic solvents may cause blood cells to aggregate without sufficient NAD^+^ extraction due to their high protein aggregation and denaturation effects. RBCs account for the majority of blood cell components in whole blood, and the NAD^+^ concentration in human RBCs [10] is equivalent to that in human whole blood [9, 11, 16, 17]. Therefore, the NAD^+^ and NMN concentrations in whole blood mainly reflect their concentrations in RBCs. In the LC–MS/MS analysis of endogenous substances, matrix effects may introduce a limitation as it is not possible to match the matrix composition of the sample and the standard. However, in our preliminary study, NAD^+^ showed a good recovery rate (low: 85.8%, high: 91.1%) via the standard addition method (ESM Table S2); thus, the matrix effect was not sensitive to NAD^+^ measurement. Furthermore, the NAD^+^ concentration in human whole blood (Table 2) approached concentrations reported previously [9, 11, 16, 17], indicating the validity of our method. The NMN concentration approached the value reported by Ito et al. [11], but it was > tenfold higher than the value reported by Okabe et al. [17]. Blood collected from healthy subjects in their 20 s to 50 s was used in our study, but the number of evaluated cases in each age group was small and the age range was narrow (we did not include individuals younger than 20 years and older than 60 years). Therefore, it is not feasible to discuss a correlation between NAD^+^ and age.

When human whole blood was stored at 4 °C, the stability of NAD^+^ increased from the initial value up to 1 week of storage, was almost the same as the initial value after 2 weeks, and decreased after 1 month (Table 3). Although it is difficult to explain this profile, the temporary increase in NAD^+^ stability may be due to the dephosphorylation of NADP under refrigerated storage, as NADP is also present in human blood [16, 18, 19]. However, NMN is less stable than NAD^+^ because it degraded by almost 50% after storage for 2 days. The reason why the stability of NMN after 1 month storage increased compared to the storage stability up to 2 weeks may be attributed to the degradation of NAD^+^ to NMN. Thus, it is difficult to maintain the NAD^+^ and NMN concentrations in the blood as in the body, and appropriate and rapid inactivation treatments will be needed. As NMN is unstable even when stored at 4 °C, the concentration may change considerably depending on the time from collection to extraction, which may explain the discrepancy among reported concentrations. In addition, there are handling issues, such as the difficulty in uniformly mixing precipitates generated during storage because whole blood is highly viscous.

The DBS sampling method is a simple sampling method, but it is limited by the inability to maintain the stability of certain target substances. Although Drolet et al. have shown that NAD^+^ can be detected via metabolomic analysis using DBS sampling, data on NAD^+^ stability are not available, and the possibility of quantification is not clear [13]. Drolet et al. used one type of DBS card (Whatman 903TM filter paper cards), but we used four types of commercially available DBS cards, including the 903 card (Table 4) [20, 21]. The main difference between the DBS cards is whether they are coated with stabilization chemicals. DMPK-A and DMPK-B are coated, whereas DMPK-C and 903 protein saver are uncoated. As only the blood extraction solution of DMPK-B was transparent and that of the others was red, it can be considered that at least hemoglobin was eluted from the filter papers other than DMPK-B (Fig. 2). DMPK-A and DMPK-B presented higher NAD^+^ and NMN stability than other DBS cards. In the same DMPK series, DMPK-C showed more than 20% lower NAD^+^ stability after storage for 1 month than DMPK-A and DMPK-B (Table S1, Fig. 3). This result indicates that coating with stabilization chemicals is effective for maintaining the storage stability of NAD^+^. DMPK-A and DMPK-B are impregnated with radical inhibitors (sodium dodecyl sulfate, tris (hydroxymethyl)aminomethane), and chaotropic agents (guanidium thiocyanate, respectively); both exert protein denaturation effects. As it is known that NAD^+^ and NMN are mainly degraded by CD38 [3, 22, 23], it is possible that these stabilization chemicals contributed to the inactivation of CD38, leading to the stabilization of NAD^+^ and NMN. DMPK-B showed higher stability than DMPK-A at both 4 °C and room temperature, suggesting that chaotropic agents are more effective in stabilizing NAD^+^ and NMN, but the differences may be derived from the impregnation concentration on each DBS card. In addition, DMPK-A and DMPK-B showed better extraction efficiency than DMPK-C and 903 protein saver (Table 5), suggesting that chemical coating also affected extraction efficiency. For example, the paper thickness is as follows: DMPK-B (744 μm) > DMPK-A (562 μm) > DMPK-C (524 μm) > 903 protein saver (509 μm), and the difference is due to the concentration of impregnated chemicals (Table 4) [21]. Therefore, this paper thickness may have acted as a physical barrier and affected the extraction efficiency. Regarding the storage temperature, the stability of NAD^+^ and NMN on all DBS cards was lower at room temperature than at 4 °C. Thus, it will be difficult to completely inactivate the enzymes involved in NAD^+^ and NMN degradation by contact with DBS cards alone, and the combination with cold preservation is more effective. However, it is also possible that the degree of adhesion of the target substance to DBS cards increases over time while the extraction rate decreases. As a result of storage stability after extraction, only DMPK-B stabilized both NAD^+^ and NMN in the blood extraction solution (100% water), whereas the other filter papers tended to increase NAD^+^ concentration and decrease NMN concentration (Table 6). The tendency to increase NAD^+^ concentration is a phenomenon observed when whole blood is refrigerated (Table 3). Conversely, the tendency to increase NAD^+^ concentration was not observed when an organic solvent was added to the extraction buffer of each filter paper and stored (67% (v/v) acetonitrile water). This result suggests that inactivation with an organic solvent is effective. The blood extraction solution of DMPK-B, which was transparent (Fig. 2), stabilized both NAD^+^ and NMN without the addition of acetonitrile, indicating that the inactivation effect in the spotting stage on the card was high compared with that on the other cards.

In the DBS method, only a part of the blood spot is usually hollowed out using a punch of a fixed size, so the homogeneity of the analytes in the blood spot is important. However, it has been reported that the volume of serum and RBCs per punch depend on the hematocrit and blood spot volume [24, 25]. Therefore, it is generally recommended that hematocrit and blood spot volume effect should be evaluated in the DBS method. In contrast, as our method hollows out the entire spot and used it for evaluation, there is no need to consider the homogeneity issue due to hematocrit and drop volume. Our method uses only 5 μL of blood and the spot size is approximately 10 mm in diameter; thus, the entire blood spot is used. We used scissors to cut out the blood spot in this study, but it will be more efficient to use a commercially available hole punch with a diameter of 10 mm or more. Most drugs and endogenous substances use plasma or serum for the concentration measurement in blood due to the localization in the plasma fraction. For such targeted substances, the DBS method, in which blood is spotted, may result in differences in DBS concentration and plasma concentration because the net serum or plasma volume may vary depending on the hematocrit in the blood. Therefore, hematocrit values may be needed to correct the DBS concentration to plasma concentration. However, our study (Table 1) and previous studies [6] show that NAD^+^ and NMN are more abundant in blood cells than in plasma. In this case, the reliability of plasma or serum concentration will be low because the presence or absence of hemolysis at the time of collection may substantially affect the results. Therefore, considering the distribution profile, NAD^+^ and NMN concentration in blood should be measured using whole blood or RBCs, not plasma or serum, and thus, our DBS method will be suitable.

Although several biomarkers of aging have been reported, no standardized method for estimating aging has been established [26]. NAD^+^ is expected to function as an aging biomarker, but a simpler measurement method is required to study the correlation with other aging-related biological parameters. In the present study, we found that DMPK-B maintained NAD^+^ stability of 85% or more for at least 2 weeks at 4 °C and 1 week at room temperature using the DBS method with DMPK-B. Additionally, NAD^+^ stability in the blood extraction solution was more than 90% for almost 2 months. This method is convenient because it does not require a special environment for storage and requires only 5 µL of blood to be collected, making it possible to self-collect blood. In addition, the sample spotted with DMPK-B can be treated as a non-biohazard sample due to the protein inactivation effect of the chemical coating, making this method advantageous in terms of safety. Although there is a report that the plasma NAD^+^ concentration decreases with age [2], our measurement method using whole blood, which has a higher NAD^+^ content than plasma and is not affected by sampling issues (such as hemolysis), is expected to clarify the correlation with aging-related diseases. In fact, the NAD^+^ concentration measured in human whole blood using our DBS method was much higher than that reported in human plasma [2]. In the future, it will be necessary to investigate changes in NAD^+^ in diseases, aging, exercise, surgery, and lifestyle habits.

A limitation of our study is that we compared the superiority or inferiority of DBS cards, and we did not discuss the absolute value required for storage stability. This is because the background data of NAD^+^ in blood are not sufficient, and it is not possible to set an allowable measurement error based on the correlation with pathological conditions and aging indicators, or the dynamic range. In the future, we need to clarify the tolerance of measurement errors based on the correlation with each index when background data are accumulated. When the evaluation method cannot be unified, such as when various DBS cards are used, it is necessary to consider correcting the difference in storage stability. In addition, there was a large variation in the storage stability of NMN in our study. There is a possibility that the influence of inter-measurement error was large because the NMN concentration in human blood was low, near the LLOQ. One of the solutions is to use a high-performance LC–MS/MS instrument, which is capable of more sensitive measurements. The optimization of the analytical column, such as using columns with smaller inner diameters, might also be a good way to improve the sensitivity of NMN.

## Conclusions

In this study, we showed that the DBS sampling method is effective in maintaining the storage stability of NAD^+^ and NMN, a NAD^+^ precursor, in human whole blood. In particular, DMPK-B was found to be the most suitable DBS card for NAD^+^ and NMN measurement at present, owing to its high storage stability and the fact that the extraction buffer is also stable during storage regardless of its composition. To our knowledge, there has been no report on a quantitative NAD^+^ measurement method in human whole blood that can be performed with as little as 5 μL of blood and be easily implemented at a medical clinic and at home. Our simple method, in which the sample can be stored for a certain period of time without limiting the place of collection, is the most advantage compared to existing methods, and has the potential to become the gold standard for NAD^+^ measurement in blood. It is expected to contribute to the acceleration of research regarding correlation between aging or aging-related diseases and NAD^+^ concentration in human blood.

## Supplementary Information

Below is the link to the electronic supplementary material.

Supplementary file1 (DOCX 26.2 KB)
