# Supplementary material for: Stabilization and quantitative measurement of nicotinamide adenine dinucleotide in human whole blood using dried blood spot sampling
Source: Anal Bioanal Chem. 2022 Dec 11;415(5):775–85. doi: 10.1007/s00216-022-04469-7 (PMC9741944; doi:10.1007/s00216-022-04469-7)
Supplement: Supplementary file 1 — Supplementary file1 (DOCX 26.2 KB) [file 216_2022_4469_MOESM1_ESM.docx]

**Supplementary Material**

**Stabilization and quantitative measurement of nicotinamide adenine dinucleotide in human whole blood using dried blood spot sampling**

Ryo Matsuyama^1, 2^, Tomoyo Omata^2^, Michiharu Kageyama^2^, Ryota Nakajima^1, 3^, Masanobu Kanou^1, 3^, Kei Yamana^1, 3^

^1^ *Nutraceutical Group, Healthcare New Business Division, Teijin Limited, Hino, Tokyo, Japan*

^2^ *Discovery DMPK Research Group*, *Toxicology & DMPK Research Department, Teijin Institute for Bio-medical Research, Teijin Pharma Limited, Hino, Tokyo, Japan*

^3^ *NOMON Co. Ltd.,* *Kasumigaseki, Chiyoda-ku, Tokyo, Japan*

Corresponding author: Ryo Matsuyama

Nutraceutical Group, Healthcare New Business Division, Teijin Limited, Hino, Tokyo, Japan

r.matsuyama@teijin.co.jp

**Table S1** Storage stability of nicotinamide adenine dinucleotide (NAD^+^) and nicotinamide mononucleotide (NMN) on dried blood spot (DBS) cards

1. **Storage at 4 ℃**

|  |  | Storage stability (%) | | | | | | | | |
| --- | --- | --- | --- | --- | --- | --- | --- | --- | --- | --- |
|  |  | NAD^+^ | | | |  | NMN | | | |
| DBS card | Storage period | Mean | SD | %CV | n |  | Mean | SD | %CV | n^1)^ |
| DMPK-A | 2 days | 81.5 | 10.9 | 13.3 | 4 |  | 73.1 | 20.2 | 27.7 | 4 |
|  | 2 weeks | 79.1 | 12.5 | 15.8 | 18 |  | 80.6 | 18.4 | 22.9 | 18 |
|  | 1 month | 71.8 | 20.9 | 29.1 | 4 |  | 99.9 | 41.9 | 42.0 | 4(1) |
| DMPK-B | 2 days | 80.7 | 11.0 | 13.6 | 4 |  | 95.4 | 33.1 | 34.7 | 4 |
|  | 2 weeks | 87.8 | 11.2 | 12.8 | 23 |  | 93.6 | 38.1 | 40.7 | 23(1) |
|  | 1 month | 79.8 | 13.9 | 17.5 | 9 |  | 78.6 | 40.2 | 51.1 | 9(3) |
| DMPK-C | 2 days | 70.1 | 12.2 | 17.4 | 5 |  | 68.4 | 19.3 | 28.2 | 5 |
|  | 2 weeks | 66.6 | 16.3 | 24.4 | 19 |  | 52.9 | 15.3 | 29.0 | 19(3) |
|  | 1 month | 54.3 | 8.4 | 15.4 | 5 |  | NC | NC | NC | 5(3) |
| 903 protein saver | 2 days | 74.1 | 10.5 | 14.2 | 5 |  | 60.1 | 9.3 | 15.5 | 5 |
|  | 2 weeks | 77.2 | 12.6 | 16.3 | 14 |  | 64.5 | 29.6 | 46.0 | 14(1) |
|  | 1 month | 82.6 | 11.0 | 13.4 | 5 |  | 56.3 | 16.8 | 29.8 | 5 |

^1)^ Numbers in parentheses represent the number of exclusions based on the limit of quantification (BLQ)

NC: Not calculated, as the concentration in most samples were below BLQ

Lower limit of quantification: 0.5 to 2 μM (NAD^+^), 0.25 to 1 μM (NMN)

Storage stability (%) = concentration after storage on DBS cards / concentration without cards before storage (day 0) × 100

1. **Storage at room temperature**

|  |  | Storage stability (%) | | | | | | | | |
| --- | --- | --- | --- | --- | --- | --- | --- | --- | --- | --- |
|  |  | NAD^+^ | | | |  | NMN | | | |
| DBS card | Storage period | Mean | SD | %CV | n |  | Mean | SD | %CV | n^1)^ |
| DMPK-A | 2 days | 71.6 | 7.7 | 10.7 | 8 |  | 122.7 | 46.0 | 37.5 | 8 |
|  | 1 week | NT | NT | NT | NT |  | NT | NT | NT | NT |
|  | 2 weeks | 51.6 | 5.6 | 10.9 | 5 |  | 124.2 | 49.1 | 39.5 | 5 |
| DMPK-B | 2 days | 82.0 | 9.9 | 12.0 | 13 |  | 96.5 | 40.4 | 41.8 | 13 |
|  | 1 week | 87.2 | 21.4 | 24.5 | 7 |  | 69.1 | 28.3 | 40.9 | 7 |
|  | 2 weeks | 67.6 | 4.6 | 6.7 | 9 |  | 66.6 | 39.6 | 59.6 | 9(2) |
| DMPK-C | 2 days | 55.9 | 7.1 | 12.6 | 7 |  | NC | NC | NC | 7(4) |
|  | 1 week | NT | NT | NT | NT |  | NT | NT | NT | NT |
|  | 2 weeks | 50.2 | 7.9 | 15.7 | 5 |  | NC | NC | NC | 5(5) |
| 903 protein saver | 2 days | 66.2 | 7.6 | 11.4 | 7 |  | 79.8 | 15.6 | 19.6 | 7(1) |
|  | 1 week | NT | NT | NT | NT |  | NT | NT | NT | NT |
|  | 2 weeks | 50.3 | 6.4 | 12.7 | 5 |  | NC | NC | NC | 5(5) |

^1)^ Numbers in parentheses represent the number of exclusions based on the limit of quantification (BLQ)

NC: Not calculated, as the concentrations in most samples were below BLQ

NT: not tested

Lower limit of quantification: 0.5 to 2 μM (NAD^+^), 0.25 to 1 μM (NMN)

Storage stability (%) = concentration after storage on DBS cards / concentration without cards before storage (day 0) × 100

**Table S2.** Recovery of nicotinamide adenine dinucleotide (NAD^+^) in human whole blood using the standard addition method

|  | Mean | SD | %CV |
| --- | --- | --- | --- |
| **Initial (without NAD^+^ standard addition) n=9** |  |  |  |
| Concentration (μM) | 24.94 | 4.64 | 18.6 |
| **NAD^+^ Standard Addition: Low (Amount added 0.45 nmol) n=8** |  |  |  |
| Calculated Concentration (μM) | 50.86 | 4.12 | 8.1 |
| Measured Concentration (μM) | 43.63 | 6.82 | 15.6 |
| Recovery (%) | 85.8 | 11.8 | 13.8 |
| **NAD^+^ Standard Addition: High (Amount added 1.35 nmol) n=8** |  |  |  |
| Calculated Concentration (μM) | 110.75 | 4.13 | 3.7 |
| Measured Concentration (μM) | 100.68 | 7.61 | 7.6 |
| Recovery (%) | 91.1 | 8.2 | 9.1 |

Human whole blood (12.5 μL) was added in a tube with 2.5 μL of saline containing 540 μmol/L NAD^+^ or 180 μmol/L NAD^+^ physiological saline. Samples were mixed by gentle pipetting and 5 μL was quickly spotted on a funnel filter paper No.5B (Kiriyama Glass Works Co., Tokyo, Japan). After drying for 10–15 min, the filter paper with blood spots was cut with scissors and placed into a tube with 300 μL of extraction buffer containing internal standard (IS; water in 667 nmol/L d4-NAD^+^ and 333 nmol/L d5-NMN). Samples were mixed using a micromixer (TOMY Microtube Mixer MT-400; TOMY Digital Biology Co. Ltd., Tokyo, Japan) for 30 min at room temperature to prepare a blood extraction solution. A portion of the blood extraction solution was placed into a tube with a 2-fold volume of acetonitrile, followed by mixing. Samples were centrifuged (24,250 × *g*, 10 min, 4 °C) and the supernatant was analyzed using LC-MS/MS. The recovery was calculated as follows:

Recovery (%) = Measured Concentration / Calculated Concentration × 100
